# Nanoconfined Methane Storage Mechanism in Deep Coal Seams: A Wettability-Coupled Simplified Local Density Model

**DOI:** 10.3390/nano15241892

**Published:** 2025-12-17

**Authors:** Liang Ji, Xianyue Xiong, Zhihong Nie, Zhengchao Zhang, Ming Yuan, Yang Zhang, Chengchao Xu, Xiaolong Zhao, Hongtao Yang, Chengming Zhao, Zheng Sun

**Affiliations:** 1 PetroChina Coalbed Methane Company Limited, Beijing 100028, China; jiliang3413@163.com (L.J.); xiongxianyue155@163.com (X.X.); niezhihong155@163.com (Z.N.); zzc01_cbm@petrochina.com.cn (Z.Z.); ym_cbm@petrochina.com.cn (M.Y.); zyyyazj@petrochina.com.cn (Y.Z.); xcc@petrochina.com.cn (C.X.); zhaoxlqh@petrochina.com.cn (X.Z.); yht123@petrochina.com.cn (H.Y.); 2 State Key Laboratory of Fine Exploration and Intelligent Development of Coal Resources, China University of Mining and Technology, Xuzhou 221116, China; zhaocm0612@163.com

**Keywords:** modified EoS, wettability-dependent adsorption, nanoscale, local density distribution, theoretical modeling

## Abstract

In deep coal seams, where nanopores (~2 nm) dominate, wettability effects, which govern molecule–wall interaction strength, critically control the methane storage, yet remain poorly understood. This work establishes, for the first time, a theoretical framework coupling the Simplified Local Density (SLD) model with wettability effects to systematically describe nanoconfined methane behavior. Key innovations include modifying the equation of state (EoS) by incorporating a molecule–wall interaction term, correlating the nanopore wall energy parameter and adsorption layer thickness with the interaction strength, and deriving wettability-dependent shifted critical properties. This approach successfully relates the local methane density distribution to the surface contact angle, bridging the knowledge gap between nanoconfined behavior and both pore size and wettability. The results show that (a) the bulk-like gas proportion in deep seams exceeds 35%, far higher than in shallow seams, indicating superior development potential; (b) the bulk-like gas increases faster with pressure than adsorbed gas, while the adsorption amount decreases by up to 46%, as the contact angle rises from 0° to 80°; (c) the modified EoS significantly impacts the bulk-like gas, reducing its amount by about 8% in 3 nm pores due to weakened intermolecular interactions. This study underscores the necessity of integrating wettability to accurately predict the nanoconfined fluid behavior, especially for deep coal seam gas.

## 1. Introduction

Development of deep coalbed methane (CBM), a newly explored unconventional gas reservoir with a depth of over 2000 m [1,2], has become a important topic in recent years because of its tremendous geological reserve and favorable production potential. In comparison with shallow coalbed methane with a depth below 800 m, the gas content inside the deep coal seams could reach as high as >25 m^3^/ton, which is much higher than <10 m^3^/ton within a shallow coal seam [3,4,5]. Meanwhile, according to the early production performance of deep CBM wells, the gas production rate declines dramatically fast first and then enters a stable stage, suggesting that the primary content for free gas is high and is estimated to reach at least 20% [6,7], which is also completely different from the shallow CBM believed to be fully water-saturated. Hence, it is urgent to shed light on the gas storage mechanism in deep coal seams, as it exhibits differences to the well-investigated shallow CBM. Advanced experiments confirmed that pores at nanoscale are dominant [8,9], mainly less than 10 nm, inside deep coal seams; therefore, nanoconfined methane behavior is the key to physically explaining the high total-gas content as well as the high free-gas content. Theoretically, at nanoscale, the interaction force induced by the nanopore wall acts as a critical role, which is neglected at macroscale, as there is a relatively restricted distance where the molecule–wall interactions can effectively affect the molecular dynamics. In essence, the molecule–wall interaction strength is closely related to the nanopore surface attributes [10,11,12], like the composition, chemical properties, roughness, and so on, in which the composition is the most prominent, as it directly determines the solid-phase atom types. Upon analysis, the nanopore surface composition in deep coal seams is complex, which mainly includes kerogen molecules with different thermal maturities and a variety of minerals, such as kaolinite, illite, montmorillonite, hydromica, etc. [13,14,15]. As the attraction force exerted by different solid phase atoms on fluid molecules varies, the fluid molecules under nanoconfinement with different surface compositions behave dramatically differently, encompassing adsorption phase and bulk-like molecules. For instance, molecular simulation dynamics (MD) and adsorption experiments have demonstrated that the absolute adsorption amount in kerogen nanopores is much higher than clay minerals with the same pore size [16,17]. Therefore, there exist varying molecule–wall interactions in deep coal seams. However, the impact of this on nanoconfined methane behavior has received limited attention; the main research currently focuses on nanoconfined fluid behavior with a relatively fixed solid phase as the nanopore wall [18,19,20]. Notably, the presence of water may occupy the adsorption sites on the nanopore wall, subsequently reducing the fluid adsorption characteristics and behavior in nanopores [21], which is not included in this research. Additionally, a critical physical parameter, surface wettability, which inherently manifests the molecule–wall interaction strength, is employed here to facilitate a clear understanding of the correlation between the molecule–wall interactions and nanoconfined methane behavior.

Efforts have been devoted to addressing the nanoconfined methane behavior with different types of nanopore walls, which can be classified as MD, experimental methods, and theoretical models in accordance with the approaches in [22,23,24]. MD is a fundamental framework established on the primary particle dynamics accounting for interactions over various atoms, acknowledged as the most suitable tool to explore the microscopic fluid behavior. As widely reported, MD has been utilized to investigate methane adsorption and transport capacity inside shale organic and inorganic nanopores [25,26], confirming that the nanopore wall type plays a critical role in affecting the nanoconfined methane behavior. Recently, the main compositions of the organic part of the shale or coal formation, kerogen molecules with a large molar weight and complex spatial structure, have been employed to simulate methane dynamics in kerogen-based nanopores [27], demonstrating the robustness of the MD. However, the majority of the MD research focused on nanoconfined methane behavior with several typical nanopore wall types, like kerogen molecule and clay minerals, has failed to capture the impact of varying molecule–wall interaction strength on the methane behavior at nanoscale. At the same time, suffering from the high computational cost and tedious preparation work prior to molecular simulation, using MD to investigate the wettability effect on methane adsorption at nanoscale is still challenging. In addition, a laboratory experiment is the direct method to measure the methane adsorption capacity of shale or coal sample, but it has two fatal deficiencies. At first, the macroscopic isotherm curve cannot be utilized to explore microscopic methane behavior; in the other words, the essential physics behind the experimental observations are difficult to determine [28,29]. Meanwhile, for the shale or coal sample, where the methane adsorption activity takes place in the nanopores, it is almost impossible to determine the nanopore wettability [30,31]. Therefore, although a lot of experiments have been performed on the gas adsorption isotherm of coal samples, they have not shed light on the wettability in terms of the microscopic methane behavior. Some useful models have been utilized to fit the measured experimental data and have been able to predict the gas adsorption performance, mainly including the Langmuir model, Freundlich model, and D-A model [32,33,34]. Notably, there models are still limited to generating macroscopic adsorption characteristics, the reliability or accuracy of which are difficult to examine. In comparison, the theoretical model based on rigorous derivation, such as the simplified local density method (SLD) and the Lattice Boltzmann method (LBM), can not only present details of nanoconfined methane behavior but can also yield macroscopic adsorption curves [35,36,37]. More importantly, the theoretical model is capable of providing instantaneous results once a desirable algorithm for the model is established, which facilitates mimicking the methane behavior at nanoscale with varying molecule–wall interaction strength. Therefore, the theoretical model is a suitable way to perform the investigation here. To our knowledge, theoretical models have mainly focused on the impact of pore size on the nanoconfined methane behavior and not on the wettability effect.

In this work, a profound theoretical framework, the simplified local density method, rooted in the molecular chemical potential equilibrium, is employed. Meanwhile, in order to extend the applicability of SLD to capture the wettability effect, the conventional equation of state (EoS) is modified by considering the gradually pronounced molecule–wall interactions as the pore size narrows into nanoscale. Moreover, the nanopore wall energy parameter (*ε_w_*) is correlated with the surface wettability in accordance with a fitted formula proposed by collecting over 30 cases of molecule–wall interaction data. In addition, regarding the reduced methane critical properties at the nanoscale confirmed by experiments and MD, formulas for the nanoconfined critical properties are derived from the modified EoS; therefore, the critical properties are related to the surface wettability as well. The adsorption phase thickness as a function of both the pore size and surface wettability is considered, contributing to an accurate calculation of the critical properties. In summary, the regular SLD framework is significantly improved by accounting for the molecule–wall interactions, detailed improvements include the modified EoS, the wettability-dependent nanopore surface properties, and the wettability-dependent nanoconfined fluid critical properties, which lay a solid background for the novelty of this research.

The paper is arranged as follows. The physical model presenting the molecule–wall interaction strength on the nanoconfined methane behavior is introduced first, highlighting the need to investigate the wettability effect. Then, the newly developed EoS is established, by considering the weakened intermolecular interactions due to the nanopore wall attraction. Critical parameters, such as methane critical properties and nanopore wall energy parameters, are successfully correlated with the surface wettability. After that, the conventional SLD framework is improved by coupling the wettability effect, and the nanoconfined methane behavior, including the adsorption behavior and bulk-like density, is related to surface wettability for the first time. A total of 78 cases of nanoconfined methane behavior with a variety of nanopore wall types from MD or experiments are employed to examine the model reliability. Then, the methane adsorption characteristics and local density distribution at nanoscale are investigated with the proposed model. Finally, several key conclusions are drawn.

## 2. Physical Model

**Figure 1:**
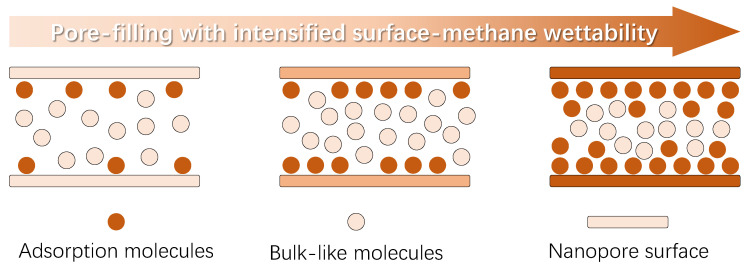
Nanoconfined fluid molecules’ behavior with increasing pore-filling due to stronger wall–molecule interactions.

Other than the main organic composition with different thermal maturity, the deep coal seam is also composed of various kinds of minerals; so, the nanopores inside deep coal exhibit a wide range of surface wettability, which is widely confirmed by the experimental results [38,39]. As illustrated in Figure 1, the physical model showing the impact of varying surface-methane wettability on the nanoconfined methane behavior is provided. To keep focus, this work emphasizes the surface wettability impact, and the other factors affecting the surface-methane interaction strength, like the surface roughness, surface chemistry, etc., will be considered in the future. First, it should be noted that the molecular behavior is inherently dominated by the intermolecular interactions. Due to the difference in the molecular attributes, particularly the atom density, the attraction force induced by the solid phase is much higher than the gas molecules, which is the essential mechanism causing the presence of the adsorption phase near the solid phase. Meanwhile, in light of the limited distance from which the interaction strength declines rapidly, the thickness of the adsorption phase is generally less than 3 molecular diameters [40,41]. Apart from the adsorption molecules, the other molecules behave similarly to the molecules at macroscopic space, which are called the bulk-like molecules here.

While the surface–molecule interaction strength is weak, few methane molecules are attracted by the nanopore walls, and the majority of the molecules confined at nanoscale are bulk-like molecules. As the surface–molecule wettability intensifies, the quantity of the adsorption molecules increases, suggesting the key impact of wettability on the methane adsorption capacity. At the same time, the behavior of bulk-like molecules is also affected by the varying surface–molecule interactions, particularly for nanopores inside the deep coal with a pore size mainly less than 5 nm. Basically, interactions between bulk-like molecules are pronounced in determining their behavior; however, this would decline to a certain extent, due to the imposed attraction force from the nanopore walls. As a result, the local density distribution for bulk-like molecules varies with different surface–molecule interactions, which is currently believed to be not relevant to the surface wettability and is commonly neglected by existed research. In short, it is urgent to investigate the wettability effect on nanoconfined methane behavior, including both the adsorption molecules and bulk-like molecules.

In order to facilitate the model establishment, some necessary assumptions are made, as listed below: (a) the nanopores inside deep coal have the geometry of nano-slits, as the most-popular observed geometries in coal and related minerals are nano-slits according to the high-resolution SEM images [42,43,44]; (b) the presence of water molecules may strongly affect the nanoconfined methane behavior, due to the invasion of fracturing fluids or original water content [45], which is not included here, as the research focuses on the wettability effect; (c) neglecting the chemical adsorption behavior that may take place between the methane and oxygen-containing functional groups, the physical adsorption mechanism dominates.

## 3. Model Establishment

Generally, the proposed model is developed by incorporating the traditional SLD framework with the wettability effect, which is described by the contact angle in the following derivations. In detail, the new EoS considering the wettability effect is introduced first, which essentially accounts for the reduced intermolecular interactions at nanoscale, due to the attractions from the nanopore walls. Then, the solid phase energy parameter and the methane critical properties are correlated with the surface wettability, suggesting the direct influences by the varying wettability, which have not been linked to the surface contact angle before.

### 3.1. Wettability Impact

The conventional EoS is presented as Equation (1), in which, term *a* represents the intermolecular interactions [46,47]. Notably, the EoS is designed to characterize the molecular behavior at a macroscopic space, which only takes care of the intermolecular interactions. All the parameters in EoS can be described as the functions of molecular basic attributes, like critical properties and the acentric factor.

(1) $$P=\frac{RT}{v_{m}-b}-\frac{a}{v_{m}(v_{m}+b)+b(v_{m}-b)}$$

(2) $$a=0.45724\frac{R^{2}T_{cb}^{2}}{P_{cb}}\alpha$$

(3) $$b=0.0778\frac{RT_{cb}}{P_{cb}}$$

(4) $$\alpha ={\left[1+(0.37464+1.54226\omega -0.26992\omega^{2})(1-\sqrt{T_{r}})\right]}^{2}$$

where *P* is the pressure, MPa; *T* is the temperature, K; *R* is the gas universal constant, 8.314 J/mol/K; *v_m_* is the molar volume, m^3^/mol; *a* and *b* represent the attraction term and repulsive term due to the intermolecular interactions, which can be obtained by Equation (2) and Equation (3), respectively; *T_cb_* and *P_cb_* are the methane critical temperature and methane critical pressure at the macroscopic space; *ω* is the methane molecular acentric factor, dimensionless; *T_r_* is the reduced temperature, dimensionless.

At the nanoscale, as analyzed, the intermolecular interactions weaken, as the nanopore wall would impose an attraction force on the bulk-like molecules. Therefore, the term *a* should reduce to calibrate this influence by the nanopore walls, manifesting the fatal flaws the conventional EoS suffers as it fails to capture this influence, which has the following expression, Equation (5), correlating the term *a* with the surface–molecule interactions [48]. It can be observed that the modified attraction term *a_eff_* would gradually approach the regular *a*, as the pore size increases, indicating that the mentioned impact would disappear with a large pore size, which aligns well with the experimental observations. Considering the modified *a*, the new EoS that can be applied to the nanoconfined molecules can be obtained.

(5) $$a_{eff}=a(1-\frac{\eta \rho_{w}\Delta w\sigma_{fw}^{2}\varepsilon_{fw}}{m\varepsilon_{f}})$$

(6) $$P=\frac{RT}{v_{m}-b}-\frac{a(1-\frac{\eta \rho_{w}\Delta w\sigma_{fw}^{2}\varepsilon_{fw}}{m\varepsilon_{f}})}{v_{m}(v_{m}+b)+b(v_{m}-b)}$$

(7) $$m=\frac{L}{2\sigma_{f}}$$

where *a_eff_* is the modified attraction term *a* for the nanoconfined EoS; *ρ_w_* is the nanopore wall atom density per volume, atoms/m^3^; △*w* is the spacing between two adjacent solid phase layers, nm; *σ_fw_* is the mixed fluid–wall molecular diameter, nm; *σ_f_* is the methane molecular diameter, nm; *ε_fw_* is the mixed fluid–wall energy parameter, J; *ε_f_* is the methane molecular energy parameter, J; *m* is the characteristic pore size, defined as the ratio of half the width to the molecular diameter, dimensionless; *η* is the fitted coefficient, which is 0.25 for the nano-slits; *L* is the width of the nano-slits, nm.

(8) $$\rho_{w}\Delta w\sigma_{fw}^{2}\varepsilon_{fw}/\varepsilon_{f}=0.65\ln(\frac{180}{\theta })$$

(9) $$P=\frac{RT}{v_{m}-b}-\frac{a(1-\frac{0.65\eta \ln(\frac{180}{\theta })}{m})}{v_{m}(v_{m}+b)+b(v_{m}-b)}$$

where *θ* is the nanopore wall–molecule contact angle, °.

In accordance with Zhao et al. (2017) [49], who revealed the mathematical relationship between the surface contact angle and the wetting parameter, as shown in Equation (8), the modified EoS can be further derived into Equation (9), which successfully relates the surface wettability with the EoS. It should be noted that the revealed relationship is mainly dependent on the interactions between organic fluids and nanopore walls with slit geometry, which is therefore suitable to describe the methane behavior in nano-slits in this work. It demonstrates that the modified EoS will degenerate into the regular EoS while the surface contact angle approaches 180°, suggesting the nanopore wall–molecule attraction force is minimal. Additionally, it is well-acknowledged that the solid phase parameters of the nanopore wall would vary with different surface wettability, and the mathematical correlation clearly presents the variation relationship. Inherently, both the nanopore wall atom density (*ρ_w_*) and the solid phase energy parameter (*ε_w_*) may increase as the wettability intensifies. For simplicity, it is believed that the atom density is only related to the nanopore wall type and remains a constant here; then, the formula correlating the energy parameter with the surface contact angle can be derived, as shown in Equation (10).

(10) $$\varepsilon_{w}=\varepsilon_{f}{\left(\frac{0.65\ln(\frac{180}{\theta })}{\rho_{w}\Delta w\sigma_{fw}^{2}}\right)}^{2}$$

where *ε_w_* is the nanopore wall energy parameter, J.

Furthermore, based on the modified EoS for nanoconfined methane (Equation (9)), the shifted critical properties at the nanoscale can be obtained from the first order and the second derivatives of pressure on molar volume [50,51], which have the following expressions. It can be observed that the methane critical properties are functions of both the pore size and surface contact angle, the reduced magnitudes of which increase with a small nanopore size, as well as a strong surface wettability [52]. In comparison, the majority of the proposed formulas currently for the nanoconfined critical properties are only related to the pore size, reflecting the advancement of this work.

(11) $$\frac{T_{cb}-T_{c}}{T_{cb}}=\frac{P_{cb}-P_{c}}{P_{cb}}=\frac{1.3\eta \sigma_{f}}{L}\ln(\frac{180}{\theta })$$

where *P_c_* and *T_c_* are the critical pressure and critical temperature under nanoconfinement, respectively.

Due to the presence of the adsorption phase, the pore space available for bulk-like methane molecules reduces, which is equivalent to the original pore size, excluding the adsorption phase thickness. As a result, the formula for the shifted critical properties becomes Equation (13) after considering the effective pore size. It can be demonstrated that the methane critical properties at the nanoscale decline more after considering the adsorption phase thickness.

(12) $$L_{eff}=L-2H_{ad}$$

(13) $$\frac{T_{cb}-T_{c}}{T_{cb}}=\frac{P_{cb}-P_{c}}{P_{cb}}=\frac{1.3\eta \sigma_{f}}{L_{eff}}\ln(\frac{180}{\theta })$$

where *H_ad_* is the adsorption phase thickness, nm; *L_eff_* is the effective nanopore width, nm.

With efforts devoted to investigating the hydrocarbon adsorption phase thickness at nanoscale, it was found that the thickness is closely related to the molecular type and pore size, as well as the surface wettability, which has the expression below [53]. The formula suggests that a small pore size and strong surface wettability contribute to the enhancement of adsorption phase thickness, which therefore may vary the methane behavior at the nanoscale inside deep coal.

(14) $$H_{ad}=(\frac{\xi }{\ln(m)}+\frac{\zeta }{m})\times (1-\frac{\theta }{180})$$

(15) $$\xi =-0.00008314MW^{2}+0.020475MW+0.030886$$

(16) $$\zeta =-0.000063565MW^{2}+0.03155MW-0.58538$$

where *MW* is the molecular molar weight, which is 16 g/mol here.

In short, from the theoretical perspective, the wettability effect on the nanoconfined methane behavior is mainly characterized by three aspects. First of all, the EoS is modified by taking care of the molecule–wall interactions; a reduced *a_eff_* with intensifying surface wettability is utilized to replace the regular intermolecular attraction term *a*. Then, the nanopore wall energy parameter is described as a function of the surface–molecule contact angle, suggesting the nonlinear correlation between the surface wettability and surface energy parameter. Additionally, the shifted critical properties of the nanoconfined methane are correlated with the surface wettability, and the wettability-dependent adsorption phase thickness is coupled. After that, the mentioned wettability effect would be incorporated into the SLD framework.

### 3.2. Modified SLD Framework

The SLD framework is rooted in the chemical potential equilibrium in a connected porous medium with multiple pore scales [54,55], as molecules with high chemical potential tend to move to places with relatively low chemical potential. Therefore, Equation (17), representing the chemical potential at macroscopic space equal to that under nanoconfinement, persists.

(17) $$\mu_{confined}=\mu_{bulk}$$

where *μ_confined_* and *μ_bulk_* are the chemical potential of methane at the nanoscale and macroscopic space, respectively.

As illustrated in Figure 1, the nanoconfined methane is affected by both the surrounding methane molecules and the nanopore walls; as a result, the nanoconfined chemical potential are contributed by intermolecular interactions and wall–molecule interactions (See Equation (18)). In detail, the chemical potential caused by the intermolecular interactions can be determined by the fugacity at the current state and at the reference state, as presented in Equations (19) and (20). Moreover, the chemical potential by molecule–wall interactions is supposed to consider the interactions from two sides of the nano-slit, which can be described as a function of the molecular distance (*z*) in terms of the nanopore wall, as presented in Equation (21).

(18) $$\mu_{confined}=\mu_{ff}(z)+\mu_{fw}(z)$$

(19) $$\mu_{bulk}=\mu_{o}(T)+RT\ln(\frac{f_{bulk}}{f_{o}})$$

(20) $$\mu_{ff}(z)=\mu_{o}(T)+RT\ln(\frac{f_{ff}(z)}{f_{o}})$$

(21) $$\mu_{fw}(z)=N_{A}[\psi^{fw}(z)+\psi^{fw}(L-z)]$$

where *μ_ff_* is the chemical potential contributed by the intermolecular interactions; *μ_fw_* is the chemical potential contributed by the molecule–wall interactions; *μ_o_* is the methane chemical potential at a reference state; *f_bulk_* is the methane fugacity at the bulk state; *f_o_* is the methane fugacity at a reference state; *f_ff_*(*z*) is the methane fugacity contributed by intermolecular interactions; *N_A_* is the Avogadro’s constant; *ψ^fw^* is the chemical potential per methane molecule induced by the methane–wall interactions.

Substituting Equations (18)~(21) into Equation (17), the nanoconfined fugacity can be derived, which is a function of the molecular distance (*z*) and the fugacity of bulk molecules. As the bulk molecule fugacity is irrelevant to *z*, the nanoconfined molecule fugacity is sensitive to the molecular relative position, aligning with the widely-reported inhomogeneous distribution inside nanopores.

(22) $$f_{ff}(z)=f_{bulk}\exp[-\frac{\psi^{fw}(z)+\psi^{fw}(L-z)}{k_{B}T}]$$

(23) $$\psi^{fw}(z)=4\pi \rho_{atom}\varepsilon_{fw}\sigma_{fw}^{2}(\frac{\sigma_{fw}^{10}}{5{\left(z+\frac{\sigma_{w}}{2}\right)}^{10}}-\frac{1}{2}\sum_{i=1}^{4}\frac{\sigma_{fw}^{4}}{{\left[z+\frac{\sigma_{w}}{2}+(i-1)\sigma_{w}\right]}^{4}})$$

where *k_B_* is the Boltzmann constant, J/K; *ρ_atom_* is the nanopore wall atom density per plane, atoms/m^2^.

The standard equation of state (EoS) presented in Equation (1), which is intended to describe the behavior of bulk molecules, can be further expressed in terms of the bulk methane density, as shown in Equation (24). Once the temperature and pressure are established, the fugacity of the bulk molecules can be determined.

(24) $$\ln\frac{f_{bulk}}{P}=\frac{b\rho_{bulk}}{1-b\rho_{bulk}}-\frac{a\rho_{bulk}}{PT(1+2b\rho_{bulk}-b^{2}\rho_{bulk}^{2})}-\ln(\frac{P}{RT\rho_{bulk}}-\frac{Pb}{RT})-\frac{a}{2\sqrt{2}RT}\ln[\frac{1+(1+\sqrt{2})b\rho_{bulk}}{1+(1-\sqrt{2})b\rho_{bulk}}]$$

Then, based on the determined bulk molecule fugacity and Equations (22) and (23), the fugacity of the nanoconfined methane as a function of *z* can be determined subsequently.

At the same time, the fugacity of nanoconfined methane can also be calculated by the modified EoS (Equation (6)), where the attraction term (*a_confined_*) and repulsive term (*b_confined_*) have the following expressions with shifted methane critical properties coupling.

(25) $$a_{confined}=a(P_{c},T_{c})\times (1-\frac{0.65\eta \ln(\frac{180}{\theta })}{m})$$

(26) $$b_{confined}=0.0778\frac{RT_{c}}{P_{c}}$$

(27) $$\ln\frac{f_{ff}(z)}{P}=\frac{b_{confined}\rho (z)}{1-b_{confined}\rho (z)}-\frac{a_{confined}\rho (z)}{PT(1+2b_{confined}\rho (z)-b_{confined}^{2}\rho (z))}-\ln(\frac{P}{RT\rho (z)}-\frac{Pb_{confined}}{RT})-\frac{a_{confined}}{2\sqrt{2}RT}\ln[\frac{1+(1+\sqrt{2})b_{confined}\rho (z)}{1+(1-\sqrt{2})b_{confined}\rho (z)}]$$

where *a_confined_* and *b_confined_* are the attraction term and repulsive term of the modified EoS, respectively; *ρ*(*z*) is the local methane density at the nanoscale, mol/m^3^.

In analogy, Equation (27) can be employed to correlate the nanoconfined fugacity with the local methane density. At a certain *z*, as the nanoconfined fugacity at *z* is calculated by Equation (22), and there is only an unknown quantity, the local methane density at *z*, for the Equation (27). Therefore, on the basis of the proposed framework, solving Equation (27) can ultimately obtain the local density; then, the wettability effect on nanoconfined methane behavior can be revealed by varying the surface contact angle.

## 4. Model Validation

Although the proposed model is established on a fundamental SLD framework, it is necessary to clarify its reliability, particularly its applicability in nanoconfined methane behavior with various nanopore types. A total of 78 cases of nanoconfined methane adsorption characteristics with approaches including MD simulation and experiments are collected here. The nanopore surface types encompass simple carbon sheets and complex macromolecules (kerogen with different thermal maturities), as well as clay minerals, which cover a wide range of nanopore wall–molecule interaction strengths, well satisfying the key requirement to verify the model. In detail, Huang et al. (2021) employed the Lattice Boltzmann method to simulate the methane vapor–liquid coexistence behavior at the nanoscale, and a key parameter, *g_w_*, was utilized to manipulate molecule–wall interaction strength [56]. Xiong et al. (2017) combined experiments and MD to investigate methane adsorption in organic-rich shale nanopores, indicating that the methane adsorption capacity decreased in the order of kerogen > clay minerals > quartz [57]. Yang et al. (2025) utilized MD simulations to shed light on the nanopore geometry on methane adsorption with regular carbon sheets utilized, suggesting the geometry angles could promote a high methane density peak [58]. Babaei et al. (2023) explored the impact of liquid water on the nanoconfined methane adsorption, utilizing a nanoscale porous medium composed of kerogen and illite to mimic the complex shale wettability [59]. Araujo et al. (2023) used MD simulations to reveal the methane adsorption behavior in kerogen nanopores considering different kerogen types and thermal maturity, manifesting that the adsorption capacity increased in the order of Kerogen I > Kerogen II > Kerogen III [60]. Zhang et al. (2025) proposed an advanced experimental method based on NMR to measure the adsorption gas amount and free-gas amount simultaneously inside coal samples [61]. Basic data relevant to the nanopore properties are collected in Table 1. The main physical parameters utilized to clarify the developed model include the absolute adsorption amount (*n_ab_*) and the adsorption gas density (*ρ_ad_*), which can be characterized by the model with Equations (28) and (29) after determining the local density distribution by Equation (27).

(28) $$n_{ab}=A\int_{\frac{\sigma_{f}}{2}}^{\frac{\sigma_{f}}{2}+H_{ad}}\rho (z)dz$$

(29) $$\rho_{ad}=\frac{1}{H_{ad}}\int_{\frac{\sigma_{f}}{2}}^{\frac{\sigma_{f}}{2}+H_{ad}}\rho (z)dz$$

where *n_ab_* is the absolute adsorption amount per gram of the adsorbent, typically used to characterize the adsorption capacity of coal samples, mol/g; *A* is the specific surface area, m^2^/g; *ρ_ad_* is the adsorption phase density, mol/m^3^.

**Figure 2:**
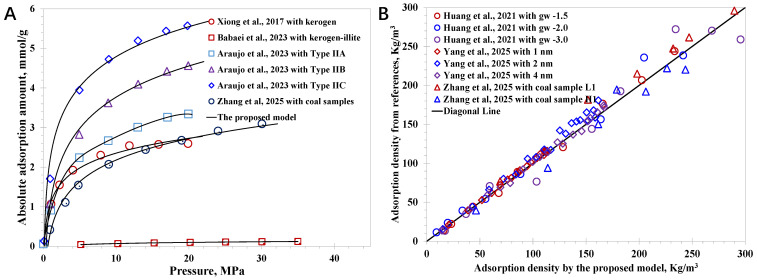
Comparisons of the model predictions with collected data: (**A**) absolute adsorption amount; (**B**) adsorption phase density [56,57,58,59,60,61].

As illustrated in Figure 2, in accordance with the comparison of the collected data to the model predictions, the model reliability can be demonstrated with an average accuracy of 3.8% for the absolute adsorption amount and 5.3% for the adsorption phase density. It indicates that the proposed model extends the classic SLD framework to capture the wettability effect. Other than that, there are three main limitations that cause the model’s inaccuracy. First of all, the proposed model considers that the nanopore surface wettability in a single case is uniform, which deviates from the realistic case that the nanopores inside a shale sample or coal sample may contain several kinds of minerals, leading to heterogeneous wettability [62]. Second, in the model establishment, some fitted critical correlations, including Equation (8) for the wetting parameter versus the surface wettability and Equation (12) for the adsorption phase thickness, are employed, which have some fitted errors, although they capture the variation tendency successfully. Additionally, nanopore surface roughness or geometry can result in deviations from the model predictions [63,64], particularly for the case with a very small pore size, as it was confirmed that the presence of geometry angles benefits methane accumulation. In short, the established model is a robust framework that can be employed to simulate methane behavior at nanoscale with different surface wettability, including kerogen nanopores with different types and thermal maturities, various clay minerals, and quartz. Meanwhile, the model accuracy and applicability can be further improved by dealing with the identified limitations, and continuous research will be performed in the future.

## 5. Results and Discussion

Methane behavior at the nanoscale is closely related to the geological reserve evaluation and production performance of the deep coalbed methane. In accordance with the proposed model, nanoconfined methane behavior is explored, particularly its varying characteristics over different surface wettability. Additionally, the influence of the modified EoS, reflecting the reduced intermolecular interaction strength, due to the presence of molecule–wall interactions, on the local methane density distribution is revealed. In the analysis below, the widely-reported parameter ranges of deep coal are utilized, including a pore size ranging from 2 to 5 nm, a pressure ranging from 10 to 25 MPa, as well as a contact angle ranging from 0° to 80°.

### 5.1. Methane Adsorption Characteristics

**Figure 3:**
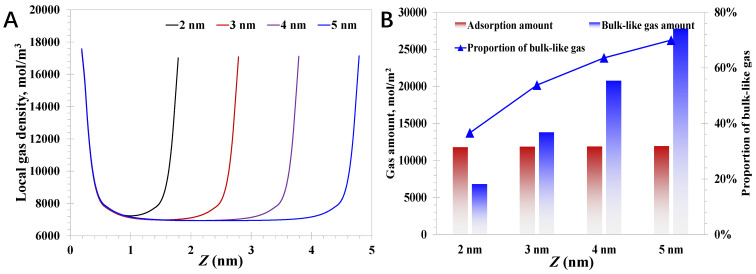
Nanoconfined methane behavior with different pore sizes: (**A**) local methane density; (**B**) contributions of adsorption gas and bulk-like gas.

The impact of the pore size is investigated first, and the pressure and contact angle are 15 MPa and 60°, respectively. As presented in Figure 3A, the typical two-peak feature of the local density distribution can be observed, indicating the evident adsorption phase, due to the strong attraction force exerted by the nanopore walls. Specifically, the adsorption phase density is almost the same within different pore sizes, indicating that the adsorption phase is nearly independent of the pore size, which aligns well with the theoretical essence that the formation of adsorption phase is mainly promoted by the nanopore wall properties. The pore size cannot affect the adsorption phase density unless the pore size is small enough that the adsorption molecules would be affected by the nanopore walls, rather than a single nanopore wall near the molecules. In terms of the production performance of deep coalbed methane wells, the existence state of the methane inside the nanopores is crucial, as the adsorption molecules adhere tightly at the nanopore wall, and their movability is fairly weak. In comparison, the bulk-like gas molecules are much less affected by the nanopore walls, which therefore move faster than the adsorption gas. In short, the greater the proportion of bulk-like gas amount, the more desirable the development efficiency of the deep coalbed methane will be. As shown in Figure 3B, the proportion of the bulk-like gas increases from 36.6% to 69.9% as the pore size enlarges; the basic formulas are provided in Equations (30)~(32). The results indicate the large nanopores expect to share a better gas movability due to the relatively high proportion of the bulk-gas amount. At the same time, it should be noted that the small nanopores have a high proportion of adsorption gas, which can reach as high as >60% within 2 nm nanopores. Due to the high discrepancy in the gas proportion of different pore sizes, as presented in Figure 3B, it can be concluded that large nanopores with pore size close to 2 nm are the typical symbol for a large geological gas reserve. As reported, the deep coal seam is rich in small nanopores, and the main gas storage state is adsorption, indicating that the overall gas mobility in the deep coal matrix is actually limited. Meanwhile, due to the extremely pore size restricting the absolute permeability and the relatively low proportion of bulk-like gas limiting the gas mobility, it is necessary to take measures to promote gas desorption and enhance the proportion of the bulk-like amount, such as heated gas injection, expanding the pressure-depleted area, etc. In addition, compared to the shallow coalbed methane with tiny gas saturation at the primary state, deep coalbed methane exhibits an evident superior development potential, which is also confirmed by the current production performance, where the gas production rates from the deep coal are 1~2 orders of magnitude higher than those from the shallow coal.

(30) $$n_{adsorption}=2\int_{\frac{\sigma_{f}}{2}}^{\frac{\sigma_{f}}{2}+H_{ad}}\rho (z)dz.$$

(31) $$n_{bulk\text{-}like}=\int_{\frac{\sigma_{f}}{2}}^{L-\frac{\sigma_{f}}{2}}\rho (z)dz-n_{adsorption}.$$

(32) $$P_{bulk\text{-}like}=\frac{n_{bulk\text{-}like}}{n_{bulk\text{-}like}+n_{adsorption}}.$$

where *n_adsorption_* is the amount of gas adsorbed per nanopore space, mol/m^2^; *n_bulk-like_* is the gas amount of the bulk-like gas per nanopore space, mol/m^2^; *P_bulk-like_* is the proportion of the bulk-like gas in the nanopores, %.

**Figure 4:**
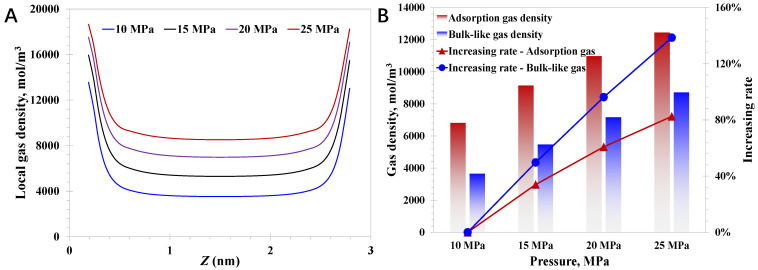
Effect of pressure on nanoconfined methane behavior: (**A**) local methane density; (**B**) adsorption density and bulk-like gas density.

Then, the pore size is set to 3 nm, and the pressure impact on the nanoconfined methane behavior is studied. As presented in Figure 4A, the local methane density enhances with increasing pressure, suggesting the high-pressure condition favors the higher total gas amount in the geological formation. Specifically, as the pressure rises from 10 MPa to 25 MPa, a rapid increase of 138.5% in the bulk-like gas density (See Equation (33)) can be observed, which is only 87.1% for the adsorption gas density. It indicates that bulk-gas density increases faster than the adsorption gas density as the pressure rises, explaining the phenomenon in which the original gas saturation in the high-pressure deep coalbed is evidently higher than the shallow coalbed. The increasing adsorption density at higher pressures is primarily due to unsaturated adsorption sites and the limited bulk-gas availability at lower pressures. As the pressure increases, the bulk-like gas molecules increase dramatically, directly enhancing the probability for the nanopore walls to capture the bulk-like gas molecules and convert them into the adsorption molecules. Notably, it can be observed that the increasing magnitude of the adsorption density versus increasing pressure gradually declines, which is due to the gradually reduced adsorption sites at a relatively high-pressure condition. In contrast, the bulk-like gas density increases linearly with the rising pressure, as presented in Figure 4B. As a result, it can be expected that the bulk-like gas density will gradually approach the adsorption gas density as the pressure continues to increase. As for the variation feature in Figure 4, it implies the effective depressurization is an efficient approach to develop both the adsorption gas and the bulk-like gas in the deep coal where nanopores are rich; therefore, large-scale reservoir stimulation that could contribute to regional pressure reduction is desirable before gas production.

(33) $$\rho_{bulk-like}=\frac{1}{L-2H_{ad}}\int_{H_{ad}}^{L-H_{ad}}\rho (z)dz$$

where *ρ_bulk-like_* is the nanoconfined bulk-like gas density, mol/m^3^.

**Figure 5:**
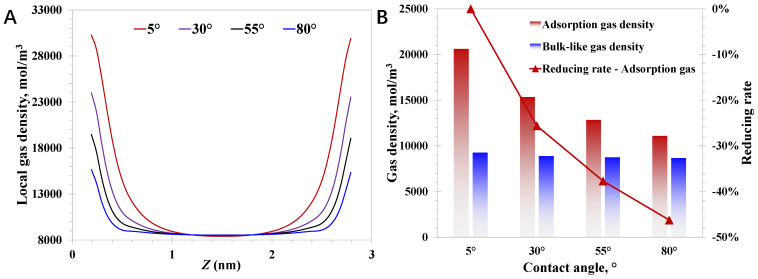
Effect of wettability (contact angle) on nanoconfined methane behavior: (**A**) aocal methane density; (**B**) adsorption density and bulk-like gas density.

After that, the pore size and pressure are set to 3 nm and 20 MPa, respectively. The nanoconfined methane behavior with the varying surface wettability is presented in Figure 5. It can be observed that the adsorption phase density is sensitive to the varying surface contact angles; Figure 5B suggests a large reduction of 46.2% in the adsorption density with the contact angle increasing from 5° to 80°. In contrast, the bulk-like gas density behaves almost the same with varying contact angles. Essentially, the adsorption activity is closely related to the molecule–wall interaction strength, resulting in the critical impact of wettability on the adsorption phase density. Additionally, regardless of the varying molecule–wall interaction strengths, the distance where the wettability can affect the local methane density generally falls in several molecule diameters. Therefore, the density of bulk-like methane molecules away from the nanopore walls is little affected by the wettability effect. Due to the dramatical wettability effect on the adsorption methane amount, it is crucial to consider the ununiform compositions of nanopore walls inside deep coal.

### 5.2. Potential Discrepancy by the Modified EoS

**Figure 6:**
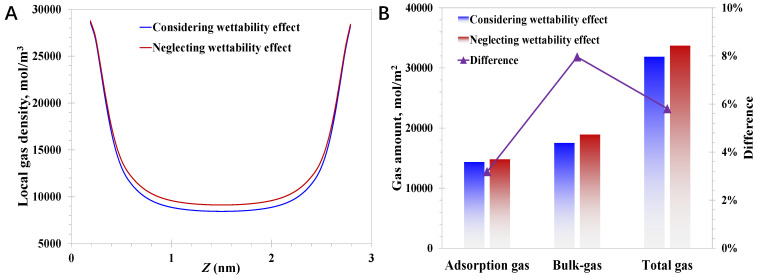
Impact of modified EoS on methane behavior in 3 nm nanopores: (**A**) local methane density; (**B**) adsorption gas and bulk-like gas amount.

In the model establishment, it can be concluded that the surface wettability effect is coupled by mainly considering the modified EoS, wettability-dependent nanopore wall energy parameter (*ε_w_*), and the wettability-dependent critical properties. As the nanopore wall energy parameter is naturally related to the surface wettability and the limited impact of the shifted critical properties has been widely investigated and reported, the research focus is on the impact of the modified EoS on the nanoconfined methane behavior here. The difference in nanoconfined methane behavior by considering or neglecting the modified EoS is examined. The pressure and surface contact angle are set to 20 MPa and 20°, respectively. As for the case of the pore size of 3 nm, it can be observed from Figure 6A that neglecting the wettability effect would lead to a rising bulk-like gas amount, the magnitude of which reaches 8.0% presented in Figure 6B. Meanwhile, little influence of neglecting the wettability on the adsorption phase amount is demonstrated, which is enhanced by only 3.2%. The mechanism behind the presented phenomenon, where neglecting the modified EoS could promote the nanoconfined gas amount, can be elucidated by the intermolecular interactions. Basically, the EoS is rooted in the intermolecular interactions, the strength of which weakens within a nanoconfined space considering the molecule–wall interactions. As a result, the gas accumulation activities that could have taken place in the bulk-like area reduce, resulting in the decline of the bulk-like gas density at the nanoscale, which aligns with the variation feature in Figure 6. Meanwhile, as the adsorption behavior is mainly controlled by molecule–wall interactions, which are much more powerful than intermolecular interactions, the reduction in the adsorption gas amount is less than the bulk-like gas amount.

**Figure 7:**
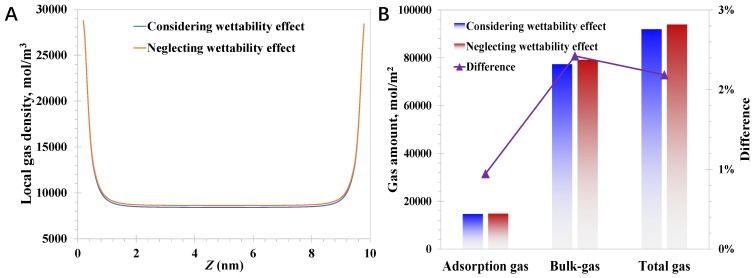
Impact of modified EoS on methane behavior in 10 nm nanopores: (**A**) local methane density; (**B**) adsorption gas and bulk-like gas amount.

While the pore size becomes 10 nm, the reduction magnitude caused by the wettability effect declines considerably compared to the case of 3 nm, which is 2.4% for the bulk-like gas amount and 0.9% for the adsorption gas amount, according to Figure 7. It indicates that the impact of wettability reduces dramatically as the pore size increases, and the variation in the magnitude of the total gas amount reduces from 5.8% for 3 nm nanopores to 2.2% for 10 nm. This phenomenon actually can be predicted by Equation (9), suggesting the modified EoS will degenerate to the regular EoS with a large pore size and weak molecule–wall interaction strength. Therefore, it can be demonstrated that the total gas amount will be overestimated with the absence of the wettability effect, which symbolizes the modified EoS only in this part. More attention should be paid to the wettability impact when the pore size narrows to ~2 nm, and the molecule–wall interactions are strong, which is important to achieve an accurate evaluation of the original reserves in deep coal seams.

## 6. Conclusions

(1) A novel wettability-coupled SLD framework for nanoconfined methane behavior is established for the first time, in which the key EoS, nanopore wall energy parameter, and shifted critical properties are correlated with the surface contact angle. A total of 78 methane adsorption cases covering a variety of nanopore compositions, indicating a wide range of molecule–wall interaction strengths, are employed to clarify the model reliability with an average accuracy of 3.8%.
(2) The high pressure of a deep coal seam is the key condition promoting the relatively high proportion of bulk-like gas amount, which reaches at least 36.6% compared to the popular <10% of the shallow coalbed methane. Due to the gradually saturated available adsorption sites as the pressure rises, the increasing magnitude of the adsorption phase density declines with higher pressure, while the bulk-like gas density almost has a linear correlation with increasing pressure.
(3) The adsorption density declines rapidly with a weakened molecule–wall interaction strength, the reduction of which is as high as 46.2%, while the contact angle increases from 5° to 80°. Neglecting the modified EoS leads to the overestimation of the gas amount at the nanoscale, as the intermolecular interaction strength reduces after considering the molecule–wall interactions, which is 8.0% for the bulk-like gas amount and 3.2% for the adsorption gas amount for 3 nm nanopores.

## Figures and Tables

**Table 1:** MD, LBM simulations, and experiments collected to examine the model reliability.

Researchers	Approach	Fluid Type	Nanopore Surface	Pore Size (nm)	Pressure (MPa)	Temperature (K)
Yang et al., 2025 [58]	MD simulations	Methane	Carbon nanopores	0.5~4.0	0~20	273~333
Zhang et al., 2025 [61]	Experiments (NMR)	Methane	Coal macromolecules	2~50	0~35	323
Saeed et al., 2023 [59]	MD simulations	Methane	Kerogen–illite	1~4	0~50	303~363
Araujo et al., 2023 [60]	MD simulations	Methane	Kerogen-I, Kerogen-II, Kerogen-III molecules	0.2~1.0	0~20	300
Huang et al., 2021 [56]	Theoretical models (LBM)	Methane	/	6~20	0~30	300
Xiong et al., 2017 [57]	Experiments, MD simulations	Methane	Clay, kerogen molecules	1~20	0~20	333

## Data Availability

The original contributions presented in this study are included in the article. Further inquiries can be directed to the corresponding author.
